# Sex differences in psychosocial functioning and neurocognition in bipolar disorder: a systematic review and meta-analysis

**DOI:** 10.1192/j.eurpsy.2025.27

**Published:** 2025-03-05

**Authors:** Maria Serra-Navarro, Derek Clougher, Vincenzo Oliva, Clàudia Valenzuela-Pascual, Michele De Prisco, María Florencia Forte, Marina Garriga, Brisa Solé, Jose Sánchez-Moreno, Norma Verdolini, Giulia Menculini, Alfonso Tortorella, Miquel Bernardo, J. Antoni Ramos-Quiroga, Anabel Martinez-Aran, Eduard Vieta, Silvia Amoretti, Carla Torrent

**Affiliations:** 1 Bipolar and Depressive Disorders Unit, Hospital Clínic de Barcelona, Fundació Clínic-Institut d’Investigacions Biomèdiques August Pi I Sunyer [IDIBAPS], CIBERSAM, ISCIII, Barcelona, Spain; 2 Departament de Medicina, Facultat de Medicina i Ciències de la Salut, Institut de Neurociències [UBNeuro], Universitat de Barcelona [UB], Barcelona, Spain; 3 BIOARABA, Department Psychiatry. Hospital Universitario de Alava. CIBERSAM. University of the Basque Country, Vitoria, Spain; 4 Local Health Unit Umbria 1, Department of Mental Health, Mental Health Center of Perugia, Perugia, Italy; 5 Department of Psychiatry, University of Perugia, Perugia, Italy; 6 Barcelona Clinic Schizophrenia Unit, Hospital Clínic de Barcelona, Departament de Medicina, Institut de Neurociències [UBNeuro], Universitat de Barcelona [UB], Institut d’Investigacions Biomèdiques August Pi I Sunyer [IDIBAPS], CIBERSAM, ISCIII, Barcelona, Spain; 7 Department of Mental Health, Hospital Universitari Vall d’Hebron, Barcelona, Catalonia, Spain; 8 Group of Psychiatry, Mental Health and Addictions, Valld’Hebron Research Institute [VHIR], Vall d’Hebron Research Institute [VHIR], Barcelona, CIBERSAM Catalonia, Spain; 9 Department of Psychiatry and Forensic Medicine, Universitat Autònoma de Barcelona, Barcelona, Catalonia, Spain

**Keywords:** bipolar disorder, meta-analysis, neurocognition, psychosocial functioning, sex

## Abstract

**Introduction:**

Impairment in both psychosocial functioning and neurocognition (NC) performance is present in bipolar disorder (BD) yet the role of sex differences in these deficits remains unclear. The present systematic review and meta-analysis examined whether males and females with BD demonstrate differences in psychosocial functioning and NC performance.

**Methods:**

The Cochrane Library, EMBASE, PsycINFO, PubMed, Scopus, and Web of Science databases were systematically searched from inception until November 20, 2023.

**Results:**

Twenty studies published between 2005 and 2023 with a total sample size of 2286 patients with BD were included. A random effects meta-analysis revealed a statistically significant result with a small effect (SMD = 0.313) for sex differences in verbal learning and memory as well as visual learning and memory (SMD = 0.263). Females outperformed males in both domains. No significant sex differences were observed for any other NC outcome or psychosocial functioning. High heterogeneity and differences in assessment scales used should be considered when interpreting these findings, given their potential impact on results.

**Conclusions:**

Future research should adopt a more homogenous, standardized approach using longitudinal designs to gain a clearer insight into sex differences in this population. This approach so may increase the use of preventative therapeutic options to address the difficult clinical challenge of reaching cognitive and functional recovery.

## Introduction

Bipolar disorder (BD) is characterized by fluctuations in mood state and is a leading cause of disability due to its cognitive and functional impact [1]. Sex differences in BD have been reported in clinical outcomes, with BD-I showing equal prevalence between sexes and BD-II being more common in females [2–4]. Females are at higher risk of depression, rapid cycling, hypomania, and a seasonal pattern [3, 5–7] whereas males more frequently experience manic episodes and substance abuse [2, 5, 6, 8].

Besides clinical outcomes, differences in neurocognition (NC) between males and females have been found. These differences are mostly in line with those detected in control participants: verbal and facial memory has been reported to be outperformed by females whereas spatial processing and motor processing by males in the general population [9, 10]. Similarly, females with BD performed better in verbal learning and memory than males [2, 5, 11]. Moreover, Carrus et al.[5] reported worse immediate memory in males with BD compared with control males and did not observe the same pattern in females. Furthermore, males with BD outperformed females with BD in attention and working memory [2, 7, 12]. Regarding processing speed, a study by Solé et al.[2] reported no differences between sexes but Gogos et al. [11] found better performance in female patients. Similarly, in semantic fluency females with BD outperformed males [11] although other studies found no differences [2, 7]. The data in Vaskinn et al. [13] and Gogos et al. [11] suggest a poorer NC performance in males compared to females, but the findings remain inconclusive. The discrepancies in the results could be explained due to different tests used to assess NC, small sample sizes, and different clinical and sociodemographic characteristics between studies.

Deficits in NC have been associated with poor psychosocial functioning [14], being verbal memory and executive function as the main predictors [15, 16]. Most of the studies have shown a better functioning profile in females in comparison with males [13, 17]. In contrast, Solé et al. [2] found no differences between sexes.

Nonetheless, results remain non-conclusive as mixed findings have been reported. As such, we conducted the present systematic review and meta-analysis to better understand these discrepancies. Understanding sex differences in cognitive functioning and functional outcomes in BD is critical for advancing both scientific knowledge and clinical practice. These differences could provide valuable insights contributing to a better understanding of their patterns in males and females, since it will enable the development of personalized interventions for this population. By tailoring interventions to address sex-specific needs, clinicians could improve both cognitive and functional outcomes, ultimately reducing the burden of the disorder on individuals and their families. To the best of our knowledge, no other study has systematically reviewed the literature exploring sex differences in psychosocial functioning and NC in BD. Specifically, the aim of the present study was to conduct a systematic review and meta-analysis to examine whether males and females with BD present differences in NC performance and psychosocial functioning. The primary question of this research is whether there are differences in neurocognitive performance and psychosocial functioning between males and females with BD. Two main hypotheses were formulated: differences will be found between males and females in cognitive performance and psychosocial functioning.

## Methods

The present systematic review and meta-analysis were conducted following the PRISMA guidelines [18] and had a registered protocol (PROSPERO-ID: CRD42022369013). The PRISMA checklist is reported in Supplementary Materials – Appendix 1.

### Selection criteria

Eligibility criteria were based on the Population, Intervention, Comparison, Outcome (PICO) framework. The following inclusion criteria were used: (1) original articles published in a peer-reviewed journal; (2) including people with BD, according to any edition of the Diagnostic and Statistical Manual for Mental Disorders (DSM) [19–21] the International Classification of Diseases (ICD) [22] the Research Diagnostic Criteria (RDC) [23]; (3) assessing and providing measures of global functioning or psychosocial functioning, self-rated or clinician-rated, or NC using validated measurement tools; and (4) comparing participants based on sex (i.e., females and males). Both observational (cross-sectional and longitudinal) and intervention studies were eligible for inclusion, but only baseline data were considered in the case of longitudinal and intervention studies. No language and age restrictions were applied. Studies were excluded if they were (1) reviews, (2) meta-analyses, (3) case reports, and (4) case series.

### Search strategy

The Cochrane Library, EMBASE, PsycINFO, PubMed, Scopus, and Web of Science databases were systematically searched from inception until November 20, 2023 (search strings are available in Supplementary Materials – Appendix 2). The backward snowballing technique was used to identify any additional papers not found in the original search.

### Procedure and data extraction

All retrieved studies were screened by title and abstract based on the previously defined inclusion and exclusion criteria and irrelevant studies were excluded. The remaining articles were then reviewed and examined at the full-text level.

Data extraction, when available, included: first author, year of publication, geographical region and country, study design, diagnostic criteria, diagnostic interview administered, study setting, total number of cases and controls (i.e., females and males), validated measurement tools used to assess outcomes, cognitive functioning measurement (specific cognitive domains evaluated, neuropsychological assessment implemented) psychosocial functioning measurement (functional evaluation and domains), type of outcome, mean and standard deviation (SD) of outcomes for females and males, mean age and SD of females and males, mean and SD of duration of BD illness for females and males, mean and SD of age of BD onset for females and males, % of BD-I among females and males, % of females and males with euthymic, depressed, hypomanic, manic, and mixed episodes, mean and SD of total, depressive, and (hypo)manic episodes number among females and males, % of females and males prescribed with psychotropic medication, psychiatric and/or medical comorbidities in females and males, instrument used to measure depressive and (hypo)manic symptoms, mean scores and SD obtained on symptom severity scale for females and males. If the data were not fully available in the published article, the corresponding authors were contacted up to two times to ask for the necessary data.

Specifically, to standardize the categorization of cognitive tests into cognitive domains, we based our approach on The International Society for Bipolar Disorders – Battery for Assessment of Neurocognition (ISBD-BANC) [24]. Overall cognitive functioning has been added to provide relevant information on general cognitive performance, reflecting global cognitive ability rather than isolated domains.

1. **Attention/vigilance**: RBANS attention/vigilance subtest – digit span and coding task [25], Wechsler Adult Intelligence Scale (WAIS-III) digit span subtest [26]; The Conners Continuous Performance Test (CPT-II) [27]; Trail Making Test Form A [28].
2. **Processing speed**: Delis–Kaplan Executive Function System (D-KEFS) [29], psychomotor speed-Trail Making subtest. It is a modification of the classic test, designed to isolate the psychomotor component [30]; The Screen for Cognitive Impairment in Psychiatry (SCIP) Processing Speed Subtest [31]; Processing speed WAIS-III [26].
3. **Executive/Working memory**: Cambridge Neuropsychological Test Automated Battery (CANTAB) Spatial Working Memory Task (SWM) Strategy [32]; Executive functioning D-KEFS subtest [29]; Stockings of Cambridge (SOC) planning and problem-solving [32]; N-back; Stroop – word and color test [33]; Wechsler Memory Scale (WMS-III) working memory sub-scale [26]; SCIP working memory subtest [31].
4. **Verbal learning and memory**: RBANS Delayed verbal memory subtest [25], California Verbal Learning Test [34] (CVLT-II) recall Trial 1 – 5; DKEFS Memory subtest [29]; RBANS – list and story learning Subtest [25]; WMS-III Auditory delayed subtest [26]; SCIP delayed verbal learning subtest [31].
5. **Visual learning and memory**: RBANS Figure recall subtest, visuo-spatial memory Spatial Recognition Memory (SRM) [25]; RBANS – figure copy and line orientation task [25]; WMS-III visual delayed WMS-III [26]; Rey – Osterrieth complex figure (ROCF) copy and recall [35].
6. **Social cognition**: face auditory ID; Pictures of Facial Affect (POFA) [36].
7. **Language:** RBANS – picture naming and semantic fluency tasks [25].
8. **Intelligence:** Wechsler Abbreviated Scale of Intelligence (WASI) [37] and Wechsler Adult Intelligence Scale (WAIS III) [26] full-scale IQ.
9. **Overall cognitive functioning**: RBANS [25], DKEF-S [29], and SCIP [31] total scores.
  When multiple cognitive measures were reported within a domain, the following strategies were applied to ensure consistency and comparability: (1) aggregation, if multiple measures originated from the same scale but no composite or total score was provided, aggregated scores were calculated using weighted averages of the raw scores, with weights based on sample sizes and (2) selection, if multiple different measures were reported, the most viable measure was selected based on its relevance, frequency of use in the literature, and comparability to other included studies.

Three authors (MSN, DC, CV) independently conducted all described stages. When a consensus was not reached, discrepancies were reached in a consensus meeting with two fellow authors (SA, CT).

### Quality appraisal

The risk of bias was assessed independently by three authors (MSN, DC, CV), and disagreements were resolved by involving two senior authors (SA, CT). The Newcastle–Ottawa Scale (NOS) [38] was used, and the scores obtained were converted according to the “Agency for Healthcare Research and Quality” (AHRQ) standards as done in Oliva et al.[39].

### Statistical analyses

Statistical analyses were conducted using *R* version 4.1.2 (R Core Team, 2020) and the separate meta-analyses for each outcome were performed via the metafor *R*-package [40] using a random-effect model (restricted maximum-likelihood estimator) [41]. Standardized mean differences (SMD) with 95% confidence intervals (CI) represented by Hedge’s g were used as effect sizes. Cochran’s *Q* [42], τ^2^ and *I*^2^ were used to test for heterogeneity. Prediction intervals were also estimated [43]. If high heterogeneity was detected (Cochran’s *Q p*-value <0.10 or *I*^2^ >50%), meta-regressions were conducted according to predefined predictors, including the mean age of females and males, the mean severity of depressive and (hypo)manic symptoms for females and males, and the percentage of females and males in treatment with psychotropic drugs, such as antidepressants, antipsychotics, lithium, or mood stabilizers. A leave-one-out sensitivity analysis excluding one study at a time from the main analysis was used to investigate each study’s influence on the overall effect size estimation. Publication bias was examined via funnel plots and using the Egger’s test [44] when at least 10 studies were available.

## Results

**Figure 1.**
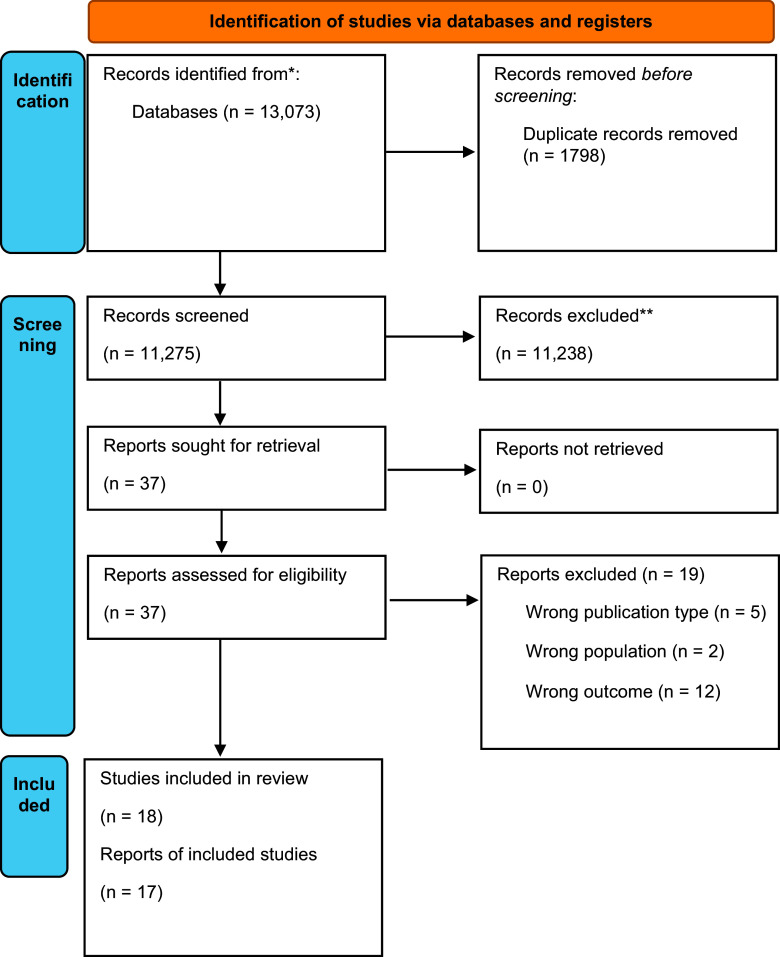
PRISMA flowchart, 2020 edition, adapted. *Consider, if feasible to do so, reporting the number of records identified from each database or register searched (rather than the total number across all databases/registers). **If automation tools were used, indicate how many records were excluded by a human and how many were excluded by automation tools.

The overall study selection process is shown in the PRISMA flowchart in Figure 1. A total of 13,073 articles were identified via a systematic search through electronic databases. Of these, 1798 duplicates were identified and removed, and 11,275 articles underwent title and abstract screening. After the exclusion of 11,238 irrelevant articles, 37 reports underwent full-text evaluation, and a total of 19 were excluded. As such, 18 studies were included in this systematic review [2, 5–7, 11, 12, 45–53] and 17 [2, 5, 6, 7, 11–13, 46–48, 49, 50, 53–57] were included in the meta-analysis. A list of excluded studies with reasons for exclusion is available in Supplementary Materials – Appendix 3.

Morgan et al. [51] was included in the systematic review due to its examination of sex-based differences in functioning among individuals with BD. However, the data were reported as percentages, rather than the continuous variables (means and standard deviations) required for our meta-analytic synthesis. Consequently, this study could not be integrated into the meta-analysis, as it lacked the necessary statistical measures for effect size estimation.

### Study characteristics

Table 1 summarizes the relevant characteristics of the 20 included studies. The studies were published between 2005 and 2023 and included a total of 2286 patients with BD. 1368 (59.8%) patients were females and 918 (40.2%) were males. The mean age of female participants was 41.5 (SD = 9.7), and the mean age of male participants was 41 (SD = 10). 19 included studies were cross-sectional [2,5,48,49,51–57,6,7,11–13,45–47] and one study was prospective [50].

**Table 1.** Characteristics of included studies

Author, year	Country	Study design	Sample characteristics	N. Females, N Males	Study setting	Age in BD sample (mean ± SD)	Primary study aim	Outcome (instrument) Neurocognitive measures Functioning measures	Diagnostic criteria	Quality of the study (NOS)
Barrett et al. [12] (2008)	Northern Ireland	Cross-sectional	26 HC, 26 BD	12 Males, 14 Females	Outpatients	Males (52.5 ± 14.1) Females (41.4; 9.1)	Examine executive function in BD and to determine how gender influences the detection of impairment when illness is in remission.	NC (COWAT, SWM, SoC, ID/ED)	DSM-IV	5/Fair
Bearden et al. [52] (2006)	USA	Cross-sectional	49 BD 38 HC	21 Males, 28 Females	Inpatients & Outpatients	Total sample (37.6 ± 11.4)	Characterize the nature of declarative memory deficits in BD and determine the relationship between clinical variables and memory function in BD.	NC (CVLT-II, WTAR, WAIS-III, TONI–3)	DSM-IV	8/Good
Blanken et al. [53] (2024)	Multicentric GAGE-BD (Netherlands; Catalonia, Spain; USA; Canada; Argentina; Brazil. Taiwan; Australia	Cross-sectional	1185 BD	540 Males, 645 Females	Outpatients	Males (64.7 ± 8.6) Females (63.4 ± 9.2)	Examine sex differences in older adults with BD and their impact on clinical outcomes, functioning and mood symptoms	Functioning (GAF)	DSM-IV	8/Good
Bücker et al. [7] (2014)	Canada	Cross-sectional	74 BD 98 HC	36 Males, 38 Females	Outpatients	Males (21.9 ± 4.00) Females (24.00 ± 4.5)	Examine healthy patterns of sex differences in cognitive functioning are altered in the early course of BD	NC (CVLT-II, CANTAB, COWAT) Functioning (GAF)	DSM-IV-TR	9/Good
Carrus et al. [5] (2010)	United Kingdom	Cross-sectional	86 BD, 46 HC	36 Males, 50 Females	Outpatients	Males (45.5 ± 12.3) Females (47.7 ± 10.3)	Examine how gender influences neurocognition identified domains which differentiate BD from HC	NC (WMS-III, WAIS-R, WCST, Hayling Sentence Completion Task)	DSM-IV	7/Good
Dittmann et al. [45] (2007)	Germany	Cross-sectional	55 BD 17 HC	26 Males, 29 Females	Outpatients	Total sample (42.3 ± 12.8)	Analyze the association between neuropsychological measures and plasma levels of homocysteine (Hcy). Explore the association between Hcy levels with age and gender and to investigate if psychosocial function is associated with cognitve impairment	NC (RBANS, TMT, LNST subtest of WAIS-III, information subtest of HAWIE-R (German version of the WAIS-R)) Functioning (SAS)	DSM-IV	8/Good
Gogos et al. [11] (2010)	Australia	Cross-sectional	38 SCZ 40 BD 43 HC	24 Males, 14 Females	Outpatients	Males (46 ± 12) Females (40 ± 11)	Examine neurocognitive deficits using RBANS comparing SCZ and BD with HC Other: to study the effects of gender on neurocognition in SCZ, BD and HC.	NC (RBANS)	DSM-IV	8/Good
Gogos et al. [46] (2023)	Australia	Cross-sectional	114 BD, 105 HC	50 Males, 64 Females	Outpatients	Males (42.5 ± 11.73) Females (35.6 ± 11.83)	Examine verbal and visual memory performance depending on sex in BD compared to controls	NC (HVLT-R, BVMT-R)	DSM-IV ICD-10	7/Good
Morgan et al. [51] (2005)	Australia	Cross-sectional	112 BD	59 Males, 53 Females	Inpatients & outpatients	Males (42) Females (43) (NO SD)	Examine the clinical and sociodemographic characteristics of individuals with BD, their levels of disability, use of medication and treatment services.	Functioning (SOFAS)	ICD-10	6/Fair
Mueser et al. [47] (2010)	USA	Cross-sectional	51 SCZ, 52 SA, 36 BD, 44 MD	10 Males, 26 Females	Outpatients	Males (58.38 ± 5.43) Females (63.46 ± 7.79)	Examine diagnostic differences and correlations of social skills in older persons with several metal illness. Explore gender differences in social skills and the relationship between social skills and neurocognitive functioning, symptoms and social contact.	NC (DKEFS, CVLT-II)	DSM-IV Axis I	9/Good
Navarra-Ventura et al. [48] (2021)	Catalonia, Spain	Cross-sectional	60 BD, 60 SCZ (30 Females, 30 Males), HC (20 Males, 20 Females)	30 Males, 30 Females	Outpatients	Males (47.5; ± 8.3) Females (46.9; ± 9.2)	Compare emotion recoginition, affective ToM, and first-and second-order cognitive ToM in BD, SCZ and HC. Examine sex-related differences in emotion recognition, affective ToM and to explore the effect of clinical variables in these social cognition subdomains.	NC (POFA, RMET)	DSM-IV-TR	6/Fair
Robb et al. [50] (1998)	Canada	Prospective	69 BD	27 Males, 42 Females	Outpatients	Total sample (36.0 ± 1.2)	Investigate gender differences in sample of BD individuals including a measure of wellbeing and funcitoning	Functioning (GAF, MOS)	Research Diagnostic Criteria	6/Fair
Sanchez-Autet et al. [49] (2018)	Spain	Cross-sectional	BD 224	78 Males, 146 Females	Outpatients	Males (45.7 ± 13.6), Females (47.8; ± 11.8)	Assess the relation of serum pro-inflammatory hepatic C-reactive protein and homocysteine levels with neurocognitive performance and psychosocial functioning and to analyze the role of gender	NC (SCIP) Functioning (FAST, GAF)	DSM-IV-TR	6/Fair
Solé et al. [2] (2022)	Spain	Cross-sectional	347 BD 115 HC	148 Males, 199 Females	Outpatients	Males (41.9, Adjusted mean 40.3 – 43.6), Females (42.4) Adjusted mean (40.9 – 43.8)	Examine sex differences in neurocognition and psychosocial functioning in BD compared to HC,	NC (WAIS (vocabulary, digit symbols coding, symbol search, arithmetic, digits and letter-number), CPT-II, TMT, CVLT, WMS-III, ROCF, WCST, SCWT, verbal and phonological fluency of the COWAT) Functioning (FAST)	DSM-IV-TR	8/Good
Suwalska & Łojko [6] (2014)	Poland	Cross-sectional	59 BD 59 HC	24 Males, 35 Females	Outpatients	Males (50 ± 10) Females (53.9 ± 10.2)	Assess the performance of lithium treated euthymic bipolar in measuring spatial working memory, planning and verbal fluency Delineate the influence of gender on cognitive functioning.	NC (TMT, FAS from the COWAT, category instant generation test, SWM, SOC)	DSM-IV	5/Fair
Tournikioti et al. [54] (2018)	Switzerland	Cross-sectional	60 BD 30 HC	Males 23, 37 Females	Inpatients & Outpatients	Median interquartil range; Males (46; 36 – 54), Females (44; 36 – 52.5),	Examine the diagnosis-specific sex effects on neurocognitive functioning (executive functions, visual memory) in BD	NC (CANTAB, SRM, PAL, SOC, ID/ED)	DSM-IV	7/Fair
Vaskinn et al. [55] (2007)	Norway	Cross-sectional	SCZ 31, BD 21, HC 31	Males 11, Females 10	Inpatients & outpatients	Total sample (38.1 ± 9.3)	Compare emotion perception in SCZ and BD, investigating the effects of gender.	Social cognition (Face auditory ID DM, face ID, Face DM, voice ID, voice DM) Functioning (Gaf-f, Gaf-s)	DSM-IV	9/Good
Vaskinn et al. [13] (2011)	Norway	Cross-sectional	SCZ 154, BD 106, HC 340	51 Males, 55 Females	Inpatients & Outpatients	Males (36.9 ± 11.2) Females (35.2 ± 10.7)	Investigate sex differences for neurocognition and social functioning in SCZ and BD. To examine the relationship between neuropsychological performance and social functioning in SCZ and BD.	NC (CVLT-II, digit symbol and digit span forward WAIS, Bergen n-back task, D-KEFS, SCWT and category fluency) Functioning (SFS)	DSM-IV	8/Good
Xu et al. [57] (2021)	China	Cross-sectional	139 BD 92 HC	44 Males, 95 Females	N/A	Medians and interquartile ranges Males (20; 18 – 23) Females 21 (18 – 23)	Examine whether deficits in neurocognition are present in first-diagnosed with patients Investigate influences of gender on neurocognitive functioning in BD	NC (RBANS, SCWT)	DSM-5	9/Good
Yazla et al. [56] 2012	Turkey	Cross-sectional	200 BD	100 Males, 100 Females	inpatient	N/A	Evaluate clinical and sociodemographic characteristics related with gender	Functioning (FSBD)	DSM-IV	5/Fair

Abbreviations: BD, Bipolar disease; HC, Healthy controls; SCZ, Schizophrenia; SA, schizoaffective disorder; NC, Neurocognition; MD, major depression. FAST, Functioning Assessment Short Test; GAF, General Assessment of Functioning; MOS, Medical Outcome Survey; POFA, Pictures of Facial Affect; RMET, Reading the Mind in the Eyes Test; SFS, Social Functioning Scale; CVLT-II, California Verbal Learning Test II; D-KEFS, Kaplan Executive Function System; WAIS, Wechsler Adult Intelligence Scale; SCWT, Stroop Color and Word Test; TMT, Trail Making Test; RBANS, the Repeatable Battery for the Assessment of Neuropsychological Status; COWAT, Control Oral Word Association test; CPT-II, Continous Performance Test-II; WMS-III, Logical Memory subtest of the Wechsler Memory Scale-III; ROCF, Rey-Osterrieth Complex Figure; WCST, Wisconsin Card Sorting Test; CANTAB, Cambridge neuropsychological test automated battery; SRM, spatial recognition memory; PAL, paired associates learning; SOC, stockings of Cambridge; Intradimensional/Extradimensional attentional set shifting (ID/ED); TONI-3, Test of Nonverbal Intelligence-3; SAS, Social Adjustment Scale; LNST, letter-number sequencing test; HVLT-R, Hopkins Verbal Learning Test-Revised; BVMT-R, Brief Visuospatial Memory Test-Revised; SOFAS. Social and Occupational Functioning Assessment Scale; FSBD, functionality scale in Bipolar Disorder.

The overall quality of the included studies was good. The average quality rating of the included studies was 7.2 (SD = 1.4; range = 5–9) (see the agreed quality grades of each study in Table 1 and a report of each general score in the Supplementary material – Appendix 4).

### Main analyses

**Figure 2.**
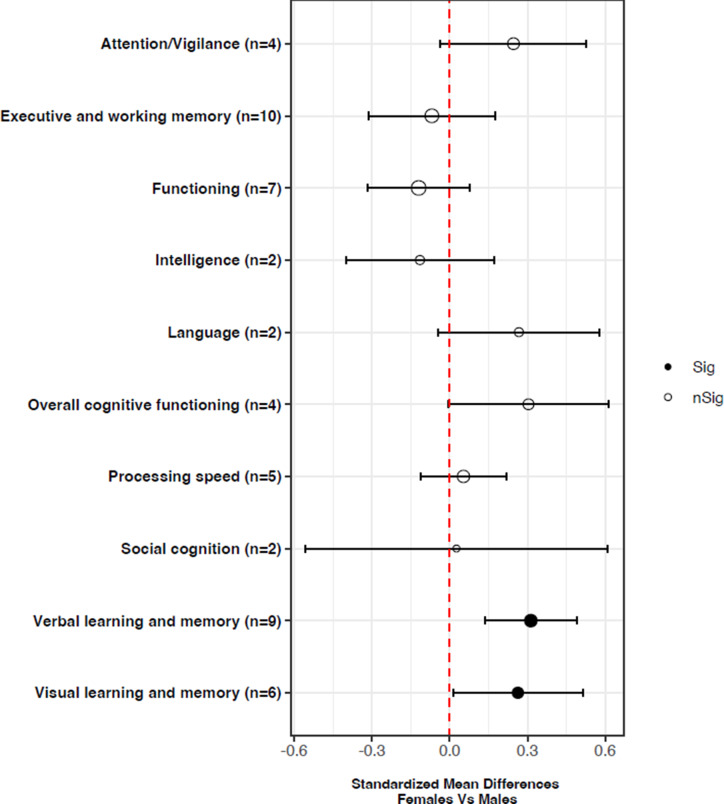
Differences in neurocognition and functioning between females (right) and males (left). Point size is proportional to the number of patients included in that specific comparison.

The main results of the meta-analyses are reported in Table 2 and Figure 2. Significant differences were found in verbal learning and memory (SMD = 0.313; 95% CI = 0.135–0.49; *p* <0.001) and visual learning and memory (SMD = 0.263; 95% CI = 0.014–0.513; *p* = 0.039), where females outperformed males in these two domains. No significant differences were found between females and males in either psychosocial functioning or any other NC outcome. Forest plots are reported in the Supplementary Materials – Appendix 5.

**Table 2.** Results of the meta-analyses in detail

Outcome type	Studies, n	Female, n	Male, n	SMD	95% CIs	*p*-value	95% PIs	*I*^2^	tau^2^	Q test *p*-value
Attention/Vigilance	4	373	259	0.246	−0.036, 0.528	0.09	−0.259, 0.751	57.99	0.05	<0.1
Executive and working memory	10	695	462	−0.069	−0.312, 0.175	0.58	−0.736, 0.599	71.41	0.1	<0.1
Functioning	7	839	617	−0.097	−0.31, 0.117	0.37	−0.607, 0.413	72.29	0.06	<0.1
Intelligence	2	105	87	−0.115	−0.4, 0.17	0.43	−0.4, 0.17	0	0	0.58
Language	2	119	60	0.267	−0.046, 0.579	0.09	−0.046, 0.579	0	0	0.36
Overall cognitive functioning	4	291	148	0.304	−0.006, 0.614	0.05	−0.215, 0.823	47.04	0.05	0.1
Processing speed	5	461	311	0.053	−0.114, 0.22	0.54	−0.174, 0.279	15.89	0.01	0.26
Social cognition	2	40	41	0.026	−0.556, 0.608	0.93	−0.744, 0.796	33.54	0.07	0.22
Verbal learning and memory	9	697	469	**0.313**	**0.135, 0.49**	**<0.001**	−0.082, 0.707	47.52	0.03	<0.1
Visual learning and memory	6	469	317	**0.263**	**0.014, 0.513**	**0.039**	−0.253, 0.78	58.83	0.05	<0.1

Abbreviations: CIs – Confidence Intervals; *I*^2^ – Higgin and Thompson’s *I*^2^ estimating of the total heterogeneity; PIs – Prediction Intervals; Qp – *p*-value for the Cochran’s Q-test of (residual) heterogeneity; SMD – Standardized mean difference; tau^2^ – between-study variance.

Note: Significant results are depicted in bold.

### Meta-regression analyses

When comparing females and males with BD, none of the predefined predictors were significantly associated with the outcomes that were significant in the main analysis. Other results of meta-regressions can be consulted in Supplementary Materials – Appendix 6.

### Sensitivity analysis

The following comparisons changed significance after the leave-one-out sensitivity analysis: (i) attention/vigilance became significant by removing the study Vaskinn et al. [13]; (ii) overall cognitive functioning became significant by removing the study Mueser et al. [47]; (iii) visual learning and memory became non-significant by removing the studies Gogos et al. [11], Tournikioti et al. [54], Xu et al. [57], Carrus et al. [5], and Gogos et al. [46]. Additional details on the sensitivity analyses are presented in the Supplementary Materials – Appendix 7.

### Publication bias

There was no evidence of publication bias (Supplementary Materials – Appendix 8).

## Discussion

To the best of our knowledge, this is the first systematic review and meta-analysis investigating sex differences in NC and psychosocial functioning in people diagnosed with BD. Two core results were found. First, significant sex differences were identified in verbal and visual memory and learning, with females performing better than males. Second, no significant sex differences were found in psychosocial functioning, although females performed better in two cognitive domains. Overall, results are of clinical importance as specific NC sex differences could be addressed to reduce impairment in patients with BD. Conversely, results suggest that psychosocial functioning may not require a specific intervention based on sex.

Regarding NC, significant sex differences were found with females performing better than males in verbal and visual memory and learning. Our findings are in line with previous studies that found sex differences in NC [9, 10], in other psychiatric populations [2, 5, 46]. Nevertheless, these results do not infer causation as to why these differences are observed. One potential explanation is that these specific sex differences are not unique to the context of mental illness as they are also present in controls without mental illness [58]. Furthermore, specific cognitive impairment can be present between patients and controls (i.e., males with BD vs. male HCs) and not be present in the opposite sex [58]. As such, we cannot conclude that the observed differences are unique to clinical populations as these impairments may have been present prior to illness onset or even due to sexual dimorphisms in brain structure [59]. In this context, we argue that studies including neuroimaging data could be important in brain anatomy and function. This may also include studies comparing the general population, high-risk population and BD in different illness stages. Further, the observed sex differences were investigated via meta-regressions using female and male age as predictor variables. While no significant differences were found, three important factors must be considered. First, a higher number of females were included in the analyses. Second, heterogeneity in the measurement of cognitive domains may also explain the lack of consistency in results regarding sex differences. Thirdly, the majority of comparisons included a very low number of studies, which may also have impacted these findings. Accordingly, we suggest that future research adopts a more homogenous approach to measuring NC in more balanced samples in terms of sex to better understand the complexity of sex differences in NC in BD.

Furthermore, the sensitivity analyses conducted provided greater insight into the significant results. Interestingly, for the visual learning and memory domain, where performance was significantly better in females, only the exclusion of Solé et al. [2] did not change the significance of the overall result. In contrast, excluding any of the other five studies rendered the result not significant. Various factors could contribute to this analysis. First, the sample size varies across studies [60]. Solé et al. [2] have the largest sample (*n* = 347) of euthymic patients with BD. Second, sample characteristics are heterogeneous with some studies only including euthymic patients [2], others symptomatic [5, 57] and the remainder a mixture of both [46, 54]. Mood state might be a major contributing factor to the differences across studies, as cognitive function tends to stabilize during euthymic phases, potentially leading to different results compared to studies with symptomatic patients. However, meta-regression analyses based on symptom severity did not change the overall results, suggesting that symptomatology alone is unlikely to explain the observed differences. Third, the illness stage also varied, for example, Xu et al. [57] focused on the early stage of the disease, and Gogos et al. [11] recruited chronic patients. Moreover, Gogos et al. [46] reported that their sample varied in terms of previous family history of BD, rapid cycling, and BD patients with comorbid anxiety disorder and substance use issues. Accordingly, the varied sample sizes and characteristics may play a significant role in the changes observed in the sensitivity analysis. Fourth, it is crucial to consider the role of medication in this analysis as research has shown that can have an impact on cognitive performance. Patients included in the present analysis were prescribed different patterns of medication (monotherapy vs. polypharmacy); some studies included patients prescribed various medications [2, 5, 11, 54], while others had samples who were only partially medicated [46] and Xu et al. [57] included non-medicated patients. Given that medication is an unavoidable confounder in clinical research [61], it is pertinent to account for these differences across studies. Additionally, an important factor to consider in the study of sex differences is the menstrual cycle together with the reproductive aging state which has been associated with worse cognitive performance according to the phase of the cycle when women are tested [62, 63]. Of the six included studies only Gogos et al. [11] collected this information. Finally, each study used different assessments of NC which most likely contributes to the changes of results in the sensitivity analysis. Overall, future studies should aim to include balanced samples and adopt a standardized approach to NC assessment while also collecting data relevant to sex differences to address limitations in the extant literature. Additionally, the identification of potential cultural variables could help to explain the sex differences.

In terms of psychosocial functioning, no significant sex differences were found. As such, our results are in line with the existing literature on other severe mental disorders such as schizophrenia [64]. However, these results do not support previous studies which highlighted NC and functional sex differences [13, 49]. The lack of consensus among studies on sex differences in functioning may partly arise from the clinical heterogeneity of BD subtypes and their associated polarity patterns. In the included studies, only three [2, 49, 53] included both BD-I and BD-II while the remaining four [7, 13, 50, 56] included BD-I only. For instance, BD-I, more evenly distributed across sexes, is often associated with manic episodes, whereas BD-II, more prevalent in females, is more linked to depressive episodes [4, 65]. Similarly, men are more likely to present hypomanic polarity whereas females are likely to present depressive polarity [66, 67]. These differences in predominant polarity could influence psychosocial functioning and cognitive performance, complicating direct comparisons across studies with mixed samples. Further research with balanced and subtype-specific cohorts is needed to disentangle these effects. Moreover, heterogeneous methods of measuring psychosocial functioning were employed. Two studies [2, 56] used the Functioning Assessment Short Test (FAST) [68], one [13] the Social Functioning Scale (SFS) [69], and four [7, 49, 50, 53] the Global Assessment of Functioning (GAF) [70]. This may explain the lack of significance observed in global psychosocial functioning and suggests that using scales, such as the FAST, that explore sub-domains of functioning could be of clinical relevance, as they provide a more comprehensive assessment of a patient’s functional abilities. This approach allows clinicians to identify specific areas of impairment and tailor interventions accordingly, leading to more effective and targeted treatment strategies. Conversely, GAF offers a single composite score which may fail to capture specific areas of strength/impairment as it is more symptom-focused. Therefore, future research should aim to explore both BD subtypes with balanced samples using standardized consensus assessment batteries approaches to measure functioning and neuropsychological performance. This approach is essential before disregarding potential sex differences, particularly important given that sub-depressive symptoms, more frequent manic episodes, and higher rates of hospitalizations are associated with functional impairment [15, 17]. This could include specific evaluation tools exploring subdomains to gain better insight into the impact of sex differences.

Overall, findings suggest that female patients with BD show better performance in both verbal and visual learning and memory compared to males with BD. Identifying the particular cognitive domains affected can inform individualized therapeutic interventions. Regarding psychosocial functioning, no significant sex differences were found. In the same line, recent findings [71] also suggest that the benefits of functional remediation (FR) do not differ by sex, indicating that tailored approaches to psychosocial functioning may not be necessary. These results emphasize that both males and females benefit similarly from FR, supporting its general applicability. Thus, the present findings must be considered in the context of the highlighted methodological challenges in the research in NC and psychosocial functioning in this population. Identifying these differences could promote preventative treatment options and offer psychotherapeutic methods to help patients reach cognitive and functional recovery, thus reducing the impact of illness on our patients. Taken as a whole, adopting sex-informed approaches to treatment may facilitate targeted therapies that optimize cognitive performance, while also acknowledging shared pathways for psychosocial improvement. This strategy may ultimately help reduce the burden of BD on patients’ lives.

The present results must be considered in light of certain limitations. Firstly, heterogeneity was observed throughout the analyses conducted. We suggest this is owed to the imbalance of sample size and the multiple different assessments used for NC and psychosocial functioning. Accordingly, we recommend a more homogenous approach that aims to standardize these inconsistencies and address limitations in the present literature. Further, a reduced number of studies provided information regarding mood state which limits the overall generalizability of the results [60]. Based on our findings, future research could significantly enhance the understanding of sex specific-factors on BD. This includes standardizing neurocognitive assessments to enable comparisons between studies, longitudinal studies to examine the evolution of sex differences over time, investigating the impact of these differences on the effectiveness of treatment options, and exploring the biological and psychosocial mechanisms underlying these disparities. Such research could refine our ability to predict outcomes and develop more tailored and effective interventions.

## Supporting information

Serra-Navarro et al. supplementary material Serra-Navarro et al. supplementary material

## Data Availability

Data are publicly available. Requests to see any data that are not included in the Article or the appendix should be directed to the corresponding author.

## Supplementary material

The supplementary material for this article can be found at http://doi.org/10.1192/j.eurpsy.2025.27.

## Additional Information

Maria Serra-Navarro and Derek Clougher are the co-first authors. PROSPERO Registration: # CRD42022369013.
