# Supplementary material for: Sex differences in psychosocial functioning and neurocognition in bipolar disorder: a systematic review and meta-analysis
Source: Eur Psychiatry. 2025 Mar 5;68(1):e45. doi: 10.1192/j.eurpsy.2025.27 (PMC12041736; doi:10.1192/j.eurpsy.2025.27)
Supplement: Serra-Navarro et al. supplementary material [file S0924933825000276sup001.doc]

**Summary supplementary materials**

[Appendix 1 - PRISMA checklist 2020 2](#__RefHeading___Toc185343355)

[Appendix 2 - Search strategy 5](#__RefHeading___Toc185343356)

[Appendix 3 - Excluded studies, with reason 11](#__RefHeading___Toc185343357)

[Appendix 4 - Quality appraisal of included studies 13](#__RefHeading___Toc185343359)

[Appendix 5 – Results, forest plots 15](#__RefHeading___Toc185343360)

[Attention/Vigilance 15](#__RefHeading___Toc185343361)

[Executive and working memory 16](#__RefHeading___Toc185343362)

[Functioning 17](#__RefHeading___Toc185343363)

[Intelligence 18](#__RefHeading___Toc185343364)

[Language 19](#__RefHeading___Toc185343365)

[Overall cognitive functioning 20](#__RefHeading___Toc185343366)

[Processing speed 21](#__RefHeading___Toc185343367)

[Social cognition 22](#__RefHeading___Toc185343368)

[Verbal learning and memory 23](#__RefHeading___Toc185343369)

[Visual learning and memory 24](#__RefHeading___Toc185343370)

[Appendix 7 – Results, leave-one-out sensitivity analyses 29](#__RefHeading___Toc185343371)

[Appendix 8 - Results, publication bias 31](#__RefHeading___Toc185343372)

# Appendix 1 - PRISMA checklist 2020

| **1Section and Topic** | **Item #** | **Checklist item** | **Location where item is reported** |
| --- | --- | --- | --- |
| **TITLE** | | |  |
| Title | 1 | Identify the report as a systematic review. | Page 1 |
| **ABSTRACT** | | |  |
| Abstract | 2 | See the PRISMA 2020 for Abstracts checklist. | Page 2 |
| **INTRODUCTION** | | |  |
| Rationale | 3 | Describe the rationale for the review in the context of existing knowledge. | Page 3 |
| Objectives | 4 | Provide an explicit statement of the objective(s) or question(s) the review addresses. | Page 4 |
| **METHODS** | | |  |
| Eligibility criteria | 5 | Specify the inclusion and exclusion criteria for the review and how studies were grouped for the syntheses. | Page 4 |
| Information sources | 6 | Specify all databases, registers, websites, organisations, reference lists and other sources searched or consulted to identify studies. Specify the date when each source was last searched or consulted. | Page 5 |
| Search strategy | 7 | Present the full search strategies for all databases, registers and websites, including any filters and limits used. | Page 5 Supp Matt |
| Selection process | 8 | Specify the methods used to decide whether a study met the inclusion criteria of the review, including how many reviewers screened each record and each report retrieved, whether they worked independently, and if applicable, details of automation tools used in the process. | Page 7 |
| Data collection process | 9 | Specify the methods used to collect data from reports, including how many reviewers collected data from each report, whether they worked independently, any processes for obtaining or confirming data from study investigators, and if applicable, details of automation tools used in the process. | Page 5 |
| Data items | 10a | List and define all outcomes for which data were sought. Specify whether all results that were compatible with each outcome domain in each study were sought (e.g. for all measures, time points, analyses), and if not, the methods used to decide which results to collect. | Page 5 |
|  | 10b | List and define all other variables for which data were sought (e.g. participant and intervention characteristics, funding sources). Describe any assumptions made about any missing or unclear information. | Page 5 |
| Study risk of bias assessment | 11 | Specify the methods used to assess risk of bias in the included studies, including details of the tool(s) used, how many reviewers assessed each study and whether they worked independently, and if applicable, details of automation tools used in the process. | Page 7 |
| Effect measures | 12 | Specify for each outcome the effect measure(s) (e.g. risk ratio, mean difference) used in the synthesis or presentation of results. | Page 7 |
| Synthesis methods | 13a | Describe the processes used to decide which studies were eligible for each synthesis (e.g. tabulating the study intervention characteristics and comparing against the planned groups for each synthesis (item #5)). | Page 7/8 |
|  | 13b | Describe any methods required to prepare the data for presentation or synthesis, such as handling of missing summary statistics, or data conversions. | Page 8 |
|  | 13c | Describe any methods used to tabulate or visually display results of individual studies and syntheses. | Page 8 |
|  | 13d | Describe any methods used to synthesize results and provide a rationale for the choice(s). If meta-analysis was performed, describe the model(s), method(s) to identify the presence and extent of statistical heterogeneity, and software package(s) used. | Page 7 |
|  | 13e | Describe any methods used to explore possible causes of heterogeneity among study results (e.g. subgroup analysis, meta-regression). | Page 7 |
|  | 13f | Describe any sensitivity analyses conducted to assess robustness of the synthesized results. | Page 7 |
| Reporting bias assessment | 14 | Describe any methods used to assess risk of bias due to missing results in a synthesis (arising from reporting biases). | Page 7 |
| Certainty assessment | 15 | Describe any methods used to assess certainty (or confidence) in the body of evidence for an outcome. | Page 7 |
| **RESULTS** | | |  |
| Study selection | 16a | Describe the results of the search and selection process, from the number of records identified in the search to the number of studies included in the review, ideally using a flow diagram. | Page 7/8  Figure 1 |
|  | 16b | Cite studies that might appear to meet the inclusion criteria, but which were excluded, and explain why they were excluded. | Page 7/8 |
| Study characteristics | 17 | Cite each included study and present its characteristics. | Table 1,  Page 8 |
| Risk of bias in studies | 18 | Present assessments of risk of bias for each included study. | Page 9 |
| Results of individual studies | 19 | For all outcomes, present, for each study: (a) summary statistics for each group (where appropriate) and (b) an effect estimate and its precision (e.g. confidence/credible interval), ideally using structured tables or plots. | Pages 7/8 Supp Mat |
| Results of syntheses | 20a | For each synthesis, briefly summarise the characteristics and risk of bias among contributing studies. | Page 7/8 |
|  | 20b | Present results of all statistical syntheses conducted. If meta-analysis was done, present for each the summary estimate and its precision (e.g. confidence/credible interval) and measures of statistical heterogeneity. If comparing groups, describe the direction of the effect. | Page 7 -8, Table 2 |
|  | 20c | Present results of all investigations of possible causes of heterogeneity among study results. | Page 7-9, Supp Mat |
|  | 20d | Present results of all sensitivity analyses conducted to assess the robustness of the synthesized results. | Page 9, Supp Mat |
| Reporting biases | 21 | Present assessments of risk of bias due to missing results (arising from reporting biases) for each synthesis assessed. | Page 8/9 Supp Mat |
| Certainty of evidence | 22 | Present assessments of certainty (or confidence) in the body of evidence for each outcome assessed. | Page 14 – 25 Supp Mat |
| **DISCUSSION** | | |  |
| Discussion | 23a | Provide a general interpretation of the results in the context of other evidence. | Page 9-12 |
|  | 23b | Discuss any limitations of the evidence included in the review. | Page 12-13 |
|  | 23c | Discuss any limitations of the review processes used. | Page 12-13 |
|  | 23d | Discuss implications of the results for practice, policy, and future research. | Page 13 |
| **OTHER INFORMATION** | | |  |
| Registration and protocol | 24a | Provide registration information for the review, including register name and registration number, or state that the review was not registered. | Page 4 |
|  | 24b | Indicate where the review protocol can be accessed, or state that a protocol was not prepared. | Page 4 |
|  | 24c | Describe and explain any amendments to information provided at registration or in the protocol. | Page 4 |
| Support | 25 | Describe sources of financial or non-financial support for the review, and the role of the funders or sponsors in the review. | Page 15-16 |
| Competing interests | 26 | Declare any competing interests of review authors. | Page 15 |
| Availability of data, code and other materials | 27 | Report which of the following are publicly available and where they can be found: template data collection forms; data extracted from included studies; data used for all analyses; analytic code; any other materials used in the review. | Page 16 |

*From:*  Page MJ, McKenzie JE, Bossuyt PM, Boutron I, Hoffmann TC, Mulrow CD, et al. The PRISMA 2020 statement: an updated guideline for reporting systematic reviews. BMJ 2021;372:n71. doi: 10.1136/bmj.n71

# Appendix 2 - Search strategy

**PUBMED**

("Bipolar Disorder"[Title/Abstract] OR "Affective Psychos*"[Title/Abstract] OR "Manic-Depressive Psychos*"[Title/Abstract] OR "Manic Depressive Psychos*"[Title/Abstract] OR "Bipolar Mood Disorder"[Title/Abstract] OR "Bipolar Depression"[Title/Abstract] OR "Manic Depression"[Title/Abstract] OR “mania” [Title/Abstract] OR “manic”[Title/Abstract] OR “bipolar disorder”[MeSH])

AND

("sex"[Title/Abstract] OR "gender"[Title/Abstract] OR "sex difference*"[Title/Abstract] OR "gender difference*"[Title/Abstract] OR "male*"[Title/Abstract] OR "female*"[Title/Abstract] OR "men"[Title/Abstract] OR "women"[Title/Abstract] OR "man"[Title/Abstract] OR "woman"[Title/Abstract] OR “sex”[MeSH])

AND

("Cognition"[Mesh] OR "Neuropsychological Tests"[Mesh] OR cognit*[Title/Abstract] OR neurocognit*[Title/Abstract] OR neuropsychol*[Title/Abstract] OR attent*[Title/Abstract] OR memory[Title/Abstract] OR execut*[Title/Abstract] OR speed[Title/Abstract] OR vigilance[Title/Abstract] OR social[Title/Abstract] OR reason*[Title/Abstract] OR functioning*[Title/Abstract] OR functionality[Title/Abstract] OR "psychosocial function*"[Title/Abstract])

**SCOPUS**

(TITLE-ABS("Bipolar Disorder") OR TITLE-ABS("Affective Psychos*") OR TITLE-ABS("Manic-Depressive Psychos*") OR TITLE-ABS("Manic Depressive Psychos*") OR TITLE-ABS("Bipolar Mood Disorder") OR TITLE-ABS("Bipolar Depression") OR TITLE-ABS("Manic Depression") OR TITLE-ABS(mania) OR TITLE-ABS(manic) OR INDEXTERMS("bipolar disorder"))

AND

(TITLE-ABS(sex) OR TITLE-ABS(gender) OR TITLE-ABS("sex difference*") OR TITLE-ABS("gender difference*") OR TITLE-ABS(male*) OR TITLE-ABS(female*) OR TITLE-ABS(men) OR TITLE-ABS(women) OR TITLE-ABS(man) OR TITLE-ABS(woman) OR INDEXTERMS(sex))

AND

(INDEXTERMS(Cognition) OR INDEXTERMS("Neuropsychological Tests") OR TITLE-ABS(cognit*) OR TITLE-ABS(neurocognit*) OR TITLE-ABS(neuropsychol*) OR TITLE-ABS(attent*) OR TITLE-ABS(memory) OR TITLE-ABS(execut*) OR TITLE-ABS(speed) OR TITLE-ABS(vigilance) OR TITLE-ABS(social) OR TITLE-ABS(reason*) OR TITLE-ABS(functioning*) OR TITLE-ABS(functionality) OR TITLE-ABS("psychosocial function*"))

**PSYCINFO**

(TI Bipolar Disorder OR TI Affective Psychos* OR TI Manic-Depressive Psychos* OR TI Manic Depressive Psychos* OR TI Bipolar Mood Disorder OR TI Bipolar Depression OR TI Manic Depression OR TI mania OR TI manic OR AB Bipolar Disorder OR AB Affective Psychos* OR AB Manic-Depressive Psychos* OR AB Manic Depressive Psychos* OR AB Bipolar Mood Disorder OR AB Bipolar Depression OR AB Manic Depression OR AB mania OR AB manic)

AND

(TI sex OR TI gender OR TI sex difference* OR TI gender difference* OR TI male* OR TI female* OR TI men OR TI women OR TI man OR TI woman OR AB sex OR AB gender OR AB sex difference* OR AB gender difference* OR AB male* OR AB female* OR AB men OR AB women OR AB man OR AB woman)

AND

(TI cognit* OR TI neurocognit* OR TI neuropsychol* OR TI attent* OR TI memory OR TI execut* OR TI speed OR TI vigilance OR TI social OR TI reason* OR TI functioning* OR TI functionality OR TI psychosocial function* OR AB cognit* OR AB neurocognit* OR AB neuropsychol* OR AB attent* OR AB memory OR AB execut* OR AB speed OR AB vigilance OR AB social OR AB reason* OR AB functioning* OR AB functionality OR AB psychosocial function*)

**Cochrane**

("Bipolar Disorder":ti,ab OR ("Affective" NEXT Psychos*):ti,ab OR ("Manic-Depressive" NEXT Psychos*):ti,ab OR ("Manic Depressive" NEXT Psychos*):ti,ab OR "Bipolar Mood Disorder":ti,ab OR "Bipolar Depression":ti,ab OR "Manic Depression":ti,ab OR mania:ti,ab OR manic:ti,ab OR [mh "bipolar disorder"])

AND

(sex:ti,ab OR gender:ti,ab OR ("sex" NEXT difference*):ti,ab OR ("gender" NEXT difference*):ti,ab OR male*:ti,ab OR female*:ti,ab OR men:ti,ab OR women:ti,ab OR man:ti,ab OR woman:ti,ab OR [mh sex])

AND

([mh Cognition] OR [mh "Neuropsychological Tests"] OR cognit*:ti,ab OR neurocognit*:ti,ab OR neuropsychol*:ti,ab OR attent*:ti,ab OR memory:ti,ab OR execut*:ti,ab OR speed:ti,ab OR vigilance:ti,ab OR social:ti,ab OR reason*:ti,ab OR functioning*:ti,ab OR functionality:ti,ab OR ("psychosocial" NEXT function*):ti,ab)

**EMBASE**

('Bipolar Disorder':ti,ab OR 'Affective Psychos*':ti,ab OR 'Manic-Depressive Psychos*':ti,ab OR 'Manic Depressive Psychos*':ti,ab OR 'Bipolar Mood Disorder':ti,ab OR 'Bipolar Depression':ti,ab OR 'Manic Depression':ti,ab OR mania:ti,ab OR manic:ti,ab OR 'bipolar disorder'/exp)

AND

(sex:ti,ab OR gender:ti,ab OR 'sex difference*':ti,ab OR 'gender difference*':ti,ab OR male*:ti,ab OR female*:ti,ab OR men:ti,ab OR women:ti,ab OR man:ti,ab OR woman:ti,ab OR sex/exp)

AND

(Cognition/exp OR 'Neuropsychological Tests'/exp OR cognit*:ti,ab OR neurocognit*:ti,ab OR neuropsychol*:ti,ab OR attent*:ti,ab OR memory:ti,ab OR execut*:ti,ab OR speed:ti,ab OR vigilance:ti,ab OR social:ti,ab OR reason*:ti,ab OR functioning*:ti,ab OR functionality:ti,ab OR 'psychosocial function*':ti,ab)

**Web of Science**

((TI="Bipolar Disorder" OR AB="Bipolar Disorder") OR (TI="Affective Psychos*" OR AB="Affective Psychos*") OR (TI="Manic-Depressive Psychos*" OR AB="Manic-Depressive Psychos*") OR (TI="Manic Depressive Psychos*" OR AB="Manic Depressive Psychos*") OR (TI="Bipolar Mood Disorder" OR AB="Bipolar Mood Disorder") OR (TI="Bipolar Depression" OR AB="Bipolar Depression") OR (TI="Manic Depression" OR AB="Manic Depression") OR (TI=mania OR AB=mania) OR (TI=manic OR AB=manic) OR ALL="bipolar disorder")

AND

((TI=sex OR AB=sex) OR (TI=gender OR AB=gender) OR (TI="sex difference*" OR AB="sex difference*") OR (TI="gender difference*" OR AB="gender difference*") OR (TI=male* OR AB=male*) OR (TI=female* OR AB=female*) OR (TI=men OR AB=men) OR (TI=women OR AB=women) OR (TI=man OR AB=man) OR (TI=woman OR AB=woman) OR ALL=sex)

AND

(ALL=Cognition OR ALL="Neuropsychological Tests" OR (TI=cognit* OR AB=cognit*) OR (TI=neurocognit* OR AB=neurocognit*) OR (TI=neuropsychol* OR AB=neuropsychol*) OR (TI=attent* OR AB=attent*) OR (TI=memory OR AB=memory) OR (TI=execut* OR AB=execut*) OR (TI=speed OR AB=speed) OR (TI=vigilance OR AB=vigilance) OR (TI=social OR AB=social) OR (TI=reason* OR AB=reason*) OR (TI=functioning* OR AB=functioning*) OR (TI=functionality OR AB=functionality) OR (TI="psychosocial function*" OR AB="psychosocial function*"))

# Appendix 3 - Excluded studies, with reason

| **Author, year** | **Title** | **doi** | **Reason for exclusion** |
| --- | --- | --- | --- |
| Andersson et al., 2008 | Neuropsychological and electrophysiological indices of neurocognitive dysfunction in bipolar II disorder | [10.1111/j.1399-5618.2008.00638.x](https://doi.org/10.1111/j.1399-5618.2008.00638.x) | Wrong outcome |
| Anselmi, 1999 | Neuropsychological patterns of memory deficits in unipolar and bipolar mood disorders (depression) | N/A | Wrong publication type |
| Bearden et al., 2001 | The neuropsychology and neuroanatomy of bipolar affective disorder: a critical review | [10.1034/j.1399-5618.2001.030302.x](https://doi.org/10.1034/j.1399-5618.2001.030302.x) | Wrong publication type |
| Boland, 2014 | Sleep disruption in cognitive and occupational functioning in Bipolar Disorder | http://dx.doi.org/10.34944/dspace/2593 | Wrong outcome |
| Borkowska & Rybakowski, 2001 | Neuropsychological frontal lobe tests indicate that bipolar depressed patients are more impaired than unipolar | [10.1034/j.1399-5618.2001.030207.x](https://doi.org/10.1034/j.1399-5618.2001.030207.x) | Wrong outcome |
| Colillas-Malet et al., 2020 | Gender differences in health-related quality of life in people with severe mental illness | <https://doi.org/10.1371/journal.pone.0229236> | Wrong outcome |
| Fellendorf et al., 2017 | Gender differences in the association between physical activity and cognitive function in individuals with bipolar disorder | 10.1016/j.jad.2017.06.048 | Wrong outcome |
| Grossman et al., 2006 | Sex differences in outcome and recovery for schizophrenia and other psychotic and nonpsychotic disorders | [10.1176/ps.2006.57.6.844](https://doi.org/10.1176/ps.2006.57.6.844) | Wrong population |
| Iosifescu et al., 2011 | Cognitive deficits are an important determinant of functional deficits in bipolar disorder: Baseline results from LiTMUS | N/A | Wrong publication type |
| Lahera et al., 2009 | Social cognition and general functioning in bipolar disorder | [10.1097/NMD.0b013e3182438eae](https://doi.org/10.1097/nmd.0b013e3182438eae) | Wrong outcome |
| Morgan et al., 2008 | Do women express and experience psychosis differently from men? epidemiological evidence from the Australian National Study of Low Pevalence (Pychotic) Disorders | <https://doi.org/10.1080/00048670701732699> | Wrong population |
| Sánchez-Moreno et al., 2018 | Factors associated with poor functional outcome in bipolar disorder: sociodemographic, clinical, and neurocognitive variables | [**https://doi.org/10.1111/acps.12894**](https://doi.org/10.1111/acps.12894) | Wrong outcome |
| Sartori et al., 2018 | Volumetric brain magnetic resonance imaging predicts functioning in bipolar disorder: A machine learning approach | [10.1016/j.jpsychires.2018.05.023](https://doi.org/10.1016/j.jpsychires.2018.05.023) | Wrong outcome |
| Taylor & Abrams, 1981 | Gender differences in bipolar affective disorder | [10.1016/0165-0327(81)90027-6](https://doi.org/10.1016/0165-0327(81)90027-6) | Wrong outcome |
| Yoldi-Negrete et al., 2018 | Predictors of functionality and quality of life in bipolar disorder | N/A | Wrong publication type |
| Zanelli et al., 2013 | Gender differences in neuropsychological performance in patients with first episode of psychosis | N/A | Wrong publication type |
| Zanelli et al., 2013 | Gender differences in neuropsychological performance across psychotic disorders--a multi-centre population based case-control study | [10.1371/journal.pone.0077318](https://doi.org/10.1371/journal.pone.0077318) | Wrong outcome |

# Appendix 4 - Quality appraisal of included studies

| **Author, year** | **Representativeness of the sample (SELECTION)** | **Sample Size (SELECTION)** | **Non-respondents (SELECTION)** | **Ascertainment of the exposure (SELECTION)** | **Comparability (COMPARABILITY)** | **Assessment of the outcome (OUTCOME)** | **Statistical Test (OUTCOME)** | **TOTAL** | **AHRQ Standards** |
| --- | --- | --- | --- | --- | --- | --- | --- | --- | --- |
| Barret et al., 2008 | 0 | 0 | 0 | 2 | 1 | 1 | 1 | 5 | Fair |
| Bearden et al. 2006 | 1 | 0 | 0 | 2 | 2 | 2 | 1 | 8 | Good |
| Blanken et al. 2024 | 1 | 1 | 0 | 2 | 1 | 2 | 1 | 8 | Good |
| Bucker et al. 2014 | 1 | 1 | 0 | 2 | 2 | 2 | 1 | 9 | Good |
| Carrus et al. 2010 | 0 | 0 | 0 | 2 | 2 | 2 | 1 | 7 | Good |
| Dittmann et al. 2007 | 1 | 0 | 0 | 2 | 2 | 2 | 1 | 8 | Good |
| Gogos et al. 2010 | 1 | 0 | 0 | 2 | 2 | 2 | 1 | 8 | Good |
| Gogos et al. 2023 | 0 | 0 | 0 | 2 | 2 | 2 | 1 | 7 | Good |
| Morgan et al. 2005 | 1 | 0 | 0 | 2 | 0 | 2 | 1 | 6 | Fair |
| Mueser et al. 2010 | 1 | 1 | 0 | 2 | 2 | 2 | 1 | 9 | Good |
| Navarra-Ventura et al. 2021 | 0 | 0 | 0 | 2 | 2 | 1 | 1 | 6 | Fair |
| Robb et al. 1998 | 1 | 0 | 0 | 2 | 0 | 2 | 1 | 6 | Fair |
| Sanchez-Autet et al. 2018 | 1 | 0 | 0 | 2 | 0 | 2 | 1 | 6 | Fair |
| Solé et al. 2022 | 1 | 0 | 0 | 2 | 2 | 1 | 1 | 8 | Good |
| Suwalska & Lojko. 2014 | 0 | 0 | 0 | 2 | 1 | 1 | 1 | 5 | Fair |
| Tournikioti et al. 2018 | 1 | 0 | 0 | 2 | 1 | 2 | 1 | 7 | Fair |
| Vaskinn et al. 2007 | 1 | 0 | 1 | 2 | 2 | 2 | 1 | 9 | Good |
| Vaskinn et al. 2011 | 1 | 0 | 0 | 2 | 2 | 2 | 1 | 8 | Good |
| Xu et al. 2021 | 1 | 0 | 1 | 2 | 2 | 2 | 1 | 9 | Good |
| Yazla et al. 2012 | 0 | 0 | 0 | 2 | 0 | 2 | 1 | 5 | Fair |

# Appendix 5 – Results, forest plots

## Attention/Vigilance

## Executive and working memory

## Functioning

## Intelligence

## Language

## Overall cognitive functioning

## Processing speed

## Social cognition

## Verbal learning and memory

## Visual learning and memory

**Appendix 6 - Results, meta-regressions**

Results in bold are significant

**Attention/vigilance**

| **Predictor** | **Number of studies** | **𝛽** | **95% CI** | **p-value** |
| --- | --- | --- | --- | --- |
| Age of females | 4 | -0.019 | -0.045, 0.007 | 0.153 |
| Age of males | 4 | -0.016 | -0.039, 0.008 | 0.193 |
| % of females taking antidepressants | 3 | **-1.196** | **-2.241, -0.152** | **0.025** |
| % of males taking antidepressants | 3 | -1.344 | -2.972, 0.285 | 0.106 |
| % of females taking antipsychotics | 3 | **-0.744** | **-1.382, -0.106** | **0.022** |
| % of males taking antipsychotics | 3 | **-0.702** | **-1.301, 0.103** | **0.022** |
| % of females taking lithium | 3 | -0.516 | -2.001, 0.969 | 0.496 |
| % of males taking lithium | 3 | -0.44 | -1.792, 0.912 | 0.524 |

**Executive and working memory**

| **Predictor** | **Number of studies** | **𝛽** | **95% CI** | **p-value** |
| --- | --- | --- | --- | --- |
| Age of females | 10 | -0.01 | -0.031, 0.01 | 0.326 |
| Age of males | 10 | -0.017 | -0.036, 0.003 | 0.091 |
| Depression severity of females | 6 | 0.031 | -0.019, 0.081 | 0.23 |
| Manic severity of females | 5 | 0.071 | -0.128, 0.272 | 0.484 |
| Depression severity of males | 6 | 0.025 | -0.019, 0.07 | 0.262 |
| Manic severity of males | 5 | 0.072 | -0.707, 0.853 | 0.854 |
| % of females taking psychotropic drugs | 5 | -0.304 | -0.724, 0.115 | 0.154 |
| % of males taking psychotropic drugs | 5 | -0.295 | -0.719, 0.129 | 0.173 |
| % of females taking antidepressants | 6 | -0.945 | -3.335, 1.445 | 0.438 |
| % of males taking antidepressants | 6 | -1.622 | -4.113, 0.869 | 0.202 |
| % of females taking antipsychotics | 7 | 0.057 | -1.109, 1.224 | 0.923 |
| % of males taking antipsychotics | 7 | -0.632 | -1.796, 0.532 | 0.287 |
| % of females taking lithium | 6 | -0.566 | -1.692, 0.56 | 0.325 |
| % of males taking lithium | 6 | -0.676 | -1.627, 0.274 | 0.163 |
| % of females taking mood stabilizers | 5 | -0.331 | -0.874, 0.211 | 0.496 |
| % of males taking mood stabilizers | 5 | -0.254 | -0.908, 0.401 | 0.447 |

**Functioning**

| **Predictor** | **Number of studies** | **𝛽** | **95% CI** | **p-value** |
| --- | --- | --- | --- | --- |
| Age of females | 4 | 0.002 | -0.027, 0.031 | 0.875 |
| Age of males | 4 | 0.005 | -0.024, 0.034 | 0.74 |
| Depression severity of females | 4 | 0.077 | -0.048, 0.202 | 0.227 |
| Manic severity of females | 4 | 0.453 | -0.201, 1.108 | 0.174 |
| Depression severity of males | 4 | 0.061 | -0.033, 0.155 | 0.205 |
| Manic severity of males | 4 | 0.668 | -0.011, 1.3477 | 0.053 |
| % of females taking antidepressants | 3 | -1.739 | -14.617, 11.139 | 0.791 |
| % of males taking antidepressants | 3 | -1.12 | -7.193, 4.952 | 0.718 |
| % of females taking antipsychotics | 4 | -1.258 | -4.102, 1.585 | 0.386 |
| % of males taking antipsychotics | 4 | 0.343 | -0.997, 1.684 | 0.616 |
| % of females taking lithium | 3 | -0.738 | -1.727, 0.251 | 0.144 |
| % of males taking lithium | 3 | -0.572 | -1.386, 0.242 | 0.169 |

**Verbal learning and memory**

| **Predictor** | **Number of studies** | **𝛽** | **95% CI** | **p-value** |
| --- | --- | --- | --- | --- |
| Age of females | 9 | -0.011 | -0.029, 0.008 | 0.258 |
| Age of males | 9 | -0.009 | -0.028, 0.01 | 0.337 |
| Depression severity of females | 6 | 0.071 | -0.023, 0.1672 | 0.141 |
| Manic severity of females | 5 | -0.088 | -0.234, 0.2341 | 0.231 |
| Depression severity of males | 6 | 0.064 | -0.019, 0.1484 | 0.1328 |
| Manic severity of males | 5 | 0.257 | -0.445, 0.959 | 0.472 |
| % of females taking psychotropic drugs | 4 | 0.136 | -0.302, 0.574 | 0.543 |
| % of males taking psychotropic drugs | 4 | 0.113 | -0.354, 0.581 | 0.634 |
| % of females taking antidepressants | 6 | 0.034 | -1.433, 1.5 | 0.964 |
| % of males taking antidepressants | 6 | 0.063 | -1.609, 1.735 | 0.941 |
| % of females taking antipsychotics | 7 | 0.06 | -0.718, 0.839 | 0.879 |
| % of males taking antipsychotics | 7 | -0.032 | -0.741, 0.677 | 0.93 |
| % of females taking lithium | 4 | -0.402 | -1.406, 0.602 | 0.433 |
| % of males taking lithium | 4 | -0.423 | -1.295, 0.449 | 0.342 |
| % of females taking mood stabilizers | 5 | 0.14 | -0.511, 0.792 | 0.673 |
| % of males taking mood stabilizers | 5 | 0.132 | -0.508, 0.771 | 0.687 |

**Visual learning and memory**

| **Predictor** | **Number of studies** | **𝛽** | **95% CI** | **p-value** |
| --- | --- | --- | --- | --- |
| Age of females | 6 | -0.001 | -0.033, 0.032 | 0.966 |
| Age of males | 6 | 0.01 | -0.019, 0.039 | 0.488 |
| % of females taking psychotropic drugs | 3 | 0.197 | -0.323, 0.717 | 0.457 |
| % of males taking psychotropic drugs | 3 | 0.227 | -0.319, 0.772 | 0.416 |
| % of females taking antidepressants | 4 | -0.094 | -1.782, 1.595 | 0.913 |
| % of males taking antidepressants | 4 | -0.275 | -2.149, 1.598 | 0.773 |
| % of females taking antipsychotics | 4 | -0.137 | -1.193, 0.919 | 0.799 |
| % of males taking antipsychotics | 4 | -0.25 | -1.219, 0.718 | 0.613 |
| % of females taking mood stabilizers | 3 | 0.216 | -0.406, 0.837 | 0.496 |
| % of males taking mood stabilizers | 3 | 0.241 | -0.399, 0.881 | 0.46 |

**Notes**: 𝛽 – regression coefficient; CI – Confidence Intervals.

# Appendix 7 – Results, leave-one-out sensitivity analyses

| **Outcome type** | **Excluded study** | **SMD** | **95% CIs** | **p-value** | **I^2^** | **tau^2^** | **Qp** |
| --- | --- | --- | --- | --- | --- | --- | --- |
| Attention/Vigilance | Gogos et al.. 2010 | 0.206 | -0.116, 0.528 | 0.21 | 68.21 | 0.05 | <0.1 |
| Attention/Vigilance | Vaskinn et al.. 2011 | **0.34** | **0.002, 0.679** | **0.049** | 59.81 | 0.05 | <0.1 |
| Attention/Vigilance | Xu et al.. 2021 | 0.109 | -0.069, 0.288 | 0.23 | 0 | 0 | 0.37 |
| Attention/Vigilance | Solé et a.. 2022 | 0.328 | -0.079, 0.735 | 0.11 | 60.22 | 0.08 | <0.1 |
| Executive and working memory | Buckner et al.. 2013 | -0.096 | -0.36, 0.167 | 0.47 | 73.31 | 0.11 | <0.1 |
| Executive and working memory | Mueser et al.. 2010 | -0.057 | -0.317, 0.203 | 0.67 | 74.67 | 0.11 | <0.1 |
| Executive and working memory | Tournikioti et al.. 2018 | -0.105 | -0.362, 0.152 | 0.42 | 72.58 | 0.1 | <0.1 |
| Executive and working memory | Vaskinn et al.. 2011 | -0.11 | -0.369, 0.149 | 0.41 | 71.2 | 0.1 | <0.1 |
| Executive and working memory | Xu et al.. 2021 | -0.105 | -0.368, 0.159 | 0.44 | 71.64 | 0.11 | <0.1 |
| Executive and working memory | Carrus et al.. 2010 | -0.05 | -0.319, 0.218 | 0.71 | 74.04 | 0.11 | <0.1 |
| Executive and working memory | Barret et al.. 2008 | -0.032 | -0.282, 0.218 | 0.8 | 72.81 | 0.1 | <0.1 |
| Executive and working memory | Sanchez-Autet et al.. 2018 | -0.072 | -0.348, 0.205 | 0.61 | 72.18 | 0.12 | <0.1 |
| Executive and working memory | Solé et a.. 2022 | 0.056 | -0.087, 0.199 | 0.45 | 0 | 0 | 0.36 |
| Executive and working memory | Suwalska and Lojko 2014 | -0.086 | -0.351, 0.179 | 0.52 | 74.24 | 0.11 | <0.1 |
| Functioning | Rob et al.. 2013 | -0.008 | -0.164, 0.147 | 0.92 | 47.38 | 0.02 | 0.11 |
| Functioning | Buckner et al.. 2013 | -0.097 | -0.345, 0.152 | 0.45 | 78.86 | 0.07 | <0.1 |
| Functioning | Vaskinn et al.. 2011 | -0.134 | -0.38, 0.112 | 0.28 | 77.42 | 0.07 | <0.1 |
| Functioning | Yazla et al.. 2012 | -0.075 | -0.325, 0.175 | 0.56 | 75.72 | 0.07 | <0.1 |
| Functioning | Sanchez-Autet et al.. 2018 | -0.097 | -0.359, 0.166 | 0.47 | 77.94 | 0.08 | <0.1 |
| Functioning | Solé et a.. 2022 | -0.156 | -0.371, 0.058 | 0.15 | 62.96 | 0.04 | <0.1 |
| Functioning | Blanken et al. 2023 | -0.138 | -0.398, 0.123 | 0.3 | 73.82 | 0.07 | <0.1 |
| Overall cognitive functioning | Gogos et al.. 2010 | 0.222 | -0.163, 0.606 | 0.26 | 61.54 | 0.07 | <0.1 |
| Overall cognitive functioning | Mueser et al.. 2010 | **0.39** | **0.109, 0.671** | **0.007** | 36.13 | 0.02 | 0.23 |
| Overall cognitive functioning | Xu et al.. 2021 | 0.198 | -0.236, 0.633 | 0.37 | 50.41 | 0.08 | 0.13 |
| Overall cognitive functioning | Sanchez-Autet et al.. 2018 | 0.331 | -0.215, 0.878 | 0.23 | 63.66 | 0.15 | <0.1 |
| Processing speed | Mueser et al.. 2010 | 0.081 | -0.1, 0.262 | 0.38 | 25.39 | 0.01 | 0.28 |
| Processing speed | Vaskinn et al.. 2011 | -0.014 | -0.171, 0.142 | 0.86 | 0 | 0 | 0.67 |
| Processing speed | Sanchez-Autet et al.. 2018 | 0.065 | -0.201, 0.331 | 0.63 | 41.53 | 0.03 | 0.15 |
| Processing speed | Solé et a.. 2022 | 0.118 | -0.11, 0.347 | 0.31 | 16.99 | 0.01 | 0.25 |
| Processing speed | Suwalska and Lojko 2014 | 0.052 | -0.152, 0.256 | 0.62 | 34.05 | 0.01 | 0.16 |
| Verbal learning and memory | Gogos et al.. 2010 | 0.301 | 0.107, 0.494 | 0.002 | 54.8 | 0.04 | <0.1 |
| Verbal learning and memory | Buckner et al.. 2013 | 0.302 | 0.096, 0.507 | 0.004 | 57.42 | 0.05 | <0.1 |
| Verbal learning and memory | Mueser et al.. 2010 | 0.353 | 0.196, 0.51 | <0.001 | 33.92 | 0.02 | 0.2 |
| Verbal learning and memory | Vaskinn et al.. 2011 | 0.266 | 0.105, 0.428 | 0.001 | 30.9 | 0.02 | <0.1 |
| Verbal learning and memory | Xu et al.. 2021 | 0.317 | 0.101, 0.532 | 0.004 | 58.64 | 0.05 | <0.1 |
| Verbal learning and memory | Carrus et al.. 2010 | 0.275 | 0.101, 0.449 | 0.002 | 40.84 | 0.02 | <0.1 |
| Verbal learning and memory | Sanchez-Autet et al.. 2018 | 0.299 | 0.077, 0.521 | 0.008 | 57.58 | 0.05 | <0.1 |
| Verbal learning and memory | Solé et a.. 2022 | 0.358 | 0.171, 0.546 | <0.001 | 36.4 | 0.03 | <0.1 |
| Verbal learning and memory | Gogos et al. 2023 | 0.33 | 0.122, 0.538 | 0.002 | 56.01 | 0.05 | <0.1 |
| Visual learning and memory | Gogos et al.. 2010 | **0.16** | **-0.038, 0.358** | **0.11** | 35.72 | 0.02 | 0.23 |
| Visual learning and memory | Tournikioti et al.. 2018 | **0.294** | **-0.01, 0.599** | **0.06** | 69.83 | 0.08 | <0.1 |
| Visual learning and memory | Xu et al.. 2021 | **0.306** | **-0.016, 0.628** | **0.06** | 68.33 | 0.09 | <0.1 |
| Visual learning and memory | Carrus et al.. 2010 | **0.292** | **-0.024, 0.608** | **0.07** | 69.79 | 0.08 | <0.1 |
| Visual learning and memory | Solé et a.. 2022 | 0.342 | 0.124, 0.561 | 0.002 | 17.55 | 0.01 | 0.16 |
| Visual learning and memory | Gogos et al. 2023 | **0.225** | **-0.06, 0.51** | **0.12** | 60.51 | 0.06 | <0.1 |

**Notes:** CIs – Confidence Intervals; I^2^ - Higgin and Thompson’s I^2^ estimating how much of the total variability in the effect size estimates can be attributed to heterogeneity among the true effects; Qp - p-value for the Cochran’s Q-test of (residual) heterogeneity; SMD – Standardized mean difference; tau^2^ - measure study estimate diversity.

Results that are different from the main analysis are reported in bold (i.e., that changed from being significant to be non-significant or vice versa).

#

# Appendix 8 - Results, publication bias

Test for Funnel Plot Asymmetry: z = 0.1433, p = 0.8860
